# Bridging vor CAR T-Zell-Therapie – eine neue Indikation der Strahlentherapie?

**DOI:** 10.1007/s00066-021-01863-5

**Published:** 2021-10-26

**Authors:** Michael Oertel, Hans Theodor Eich

**Affiliations:** Klinik für Strahlentherapie – Radioonkologie, Universitätsklinikum Münster, Albert-Schweitzer-Campus 1, Gebäude A1, 48149 Münster, Deutschland

## Hintergrund

Patient*Innen mit diffus großzelligem B‑Zell-Lymphom (DLBCL) erleiden in ca. einem Drittel der Fälle ein Rezidiv oder sind primär therapierefraktär (rezidiviert/refraktär, r/r; [1]). Als Salvage-Strategie wird eine Hochdosischemotherapie mit nachfolgender autologer Stammzelltransplantation angestrebt, die jedoch nur bei einer Minderheit der Patienten zur Heilung führt [1, 2]. Nach mindestens zwei erfolgten Therapielinien ist in Deutschland eine Behandlung mit chimären Antigenrezeptor-T-Zellen (CAR-T-Zellen) zugelassen und ermöglicht eine spezifische, immunologische Tumorantwort [3]. Beim Produkt Axicabtagen Ciloleucel (Axi-cel) werden autologe T‑Lymphozyten des Patienten/der Patientin mittels Leukapherese entnommen, ex vivo mit einer lymphomgerichteten Antikörperdomäne gegen CD19 genetisch modifiziert und anschließend reinfundiert [2]. Das Toxizitätsspektrum der CAR-T-Zell-Therapie (CAR-T-ZT) ist divers und umfasst verschiedene Organtoxizitäten und hämatologische sowie immunologische Nebenwirkungen, insbesondere die übermäßige Freisetzung inflammatorischer Zytokine (sog. Zytokin-Freisetzungssyndrom, cytokine release syndrome (CRS); [2, 4]).

Eine konditionierende Therapie („Bridging-Konzept“) zur Lymphozytendepletion mittels Radio- oder Systemtherapie senkt die Krankheitslast und kann die Wirksamkeit der CAR-T-Zellen steigern [2, 4]. Zudem kann so die Zeit zwischen Apherese und Reinfusion der CAR-T-Zellen (i. d. R. mehrere Wochen) überbrückt werden. Allerdings ist offen, ob hierdurch zusätzliche Toxizitäten auftreten und welchen Stellenwert die Radiotherapie (RT) in dieser Behandlungssituation einnimmt.

## Methode/Patientengut

In einer retrospektiven Kohortenanalyse identifizierten die Kolleg*Innen am MD Anderson Cancer Center in Houston, Texas, insgesamt 148 Patient*Innen mit r/r großzelligem B‑Zell-Lymphom und geplanter CAR-T-ZT [5]. Hiervon hatten 78 % der Patient*Innen ein DLBCL, meist in einem fortgeschrittenen Stadium: 87 % aller Patient*Innen waren bei Apherese im Stadium Ann-Arbor III/IV. Das mediane Alter betrug 60 Jahre (18–85 Jahre), 28 % der Patient*Innen waren Frauen. Im Median wurden 3 Therapielinien vor der CAR-T-ZT durchgeführt. Das mediane Follow-up lag bei 11,1 Monaten.

## Ergebnisse

Bei 55 % der Patient*Innen wurde eine „Bridging-Therapie“ (BT) vorgenommen, entweder als Systemtherapie (ST, 75 %), RT (14 %) oder als kombinierte Therapie (11 %). Die mediane Dosis betrug 35,2 Gy bzw. 35 Gy in der Gruppe der alleinigen Strahlentherapie und der kombinierten Modalität, jeweils mit einer medianen Fraktionsdosis von 2,5 Gy. Die Radiotherapie umfasste bei neun Patient*Innen alle aktiven Lymphommanifestationen und bei acht Patient*Innen nur einen Teil der betroffenen Lokalisationen (sog. fokale RT). In der Gruppe der BT gab es signifikant häufiger einen internationalen Prognose-Index (IPI) von ≥ 3 (*p*< 0,01), einen Lymphom-Bulk (*p* = 0,01), einen ECOG-Score 2–3 (*p* = 0,01) und eine erhöhte LDH (*p* < 0,01). Nach einer medianen Zeit von 29 bis 29,5 Tagen (BT vs. Non-BT) erhielten 124 Patient*Innen eine CAR-T-ZT mit Axi-cel. Die 24 Patient*Innen, die keine CAR-T-Zell-Infusion erhielten (88 % infolge einer Lymphomprogression), erreichten ein progressionsfreies (PFS) und Gesamtüberleben (OS) von 0,7 bzw. 1,3 Monaten. Insgesamt betrug das 1‑Jahres-PFS 30 % und das 1-Jahres-OS 56 %. Im Vergleich der Non-BT- und der BT-Gruppe ergab sich, bezogen auf die initiale Patientenzahl, ein signifikanter Unterschied mit 40 % bzw. 65 % in der Non-BT-Gruppe gegenüber 21 % bzw. 48 % in der BT-Gruppe für das 1‑Jahres-PFS bzw. OS (*p* = 0,01 bzw. 0,05). Für die Patient*Innen mit erfolgter CAR-T-ZT ergab sich ein 1‑Jahres-PFS und OS von 37 % und 64 % ohne signifikante Unterschiede zwischen BT und Non-BT (*p* = 0,06 bzw. *p* = 0,22).

Im Vergleich der BT-Modalitäten wiesen die nur bestrahlten Patient*Innen gegenüber der ST ein längeres PFS (8,9 Monate vs. 4,7 Monate; *p* = 0,05) und ein höheres Ansprechen auf (RT: 100 % bzw. 82 % vs. ST: 67 % bzw. 38 % bei ST für die Gesamt- und Komplettansprechrate; *p* = 0,03 bzw. *p* = 0,01) auf. Von den Patient*Innen mit fokaler RT waren 6 progredient oder rezidivierten, hiervon drei an Stellen, die bereits initial befallen waren, jedoch nicht bestrahlt wurden. Keine der untersuchten BT resultierte in einer Zunahme der Grad-3-Nebenwirkungen im Hinblick auf das CRS oder neurologische Toxizitäten. Nach CAR-T-ZT verstarben 43 der 124 Patient*Innen während des Follow-ups (32 lymphombedingt und 11 toxizitätsbedingt).

## Schlussfolgerung der Autoren

Die Bridging-Therapie (BT) scheint in der Leukapheresekohorte aufgrund eines Selektionsbias mit einem verringerten PFS und OS assoziiert zu sein; der Einfluss in der Gruppe der tatsächlich CAR-T-Zell-Therapierten ist unklar. Im Intervall zwischen Leukapherese und CAR-T-ZT ergibt sich eine signifikante lymphombedingte Letalität; bei Progress ist die Prognose infaust. Eine BT mit alleiniger Strahlentherapie erwies sich als sicher und effektiv. Hierbei scheint eine „comprehensive“ RT aller aktiven Lymphommanifestationen mit einer verbesserten Prognose einherzugehen.

## Kommentar

- Der Einsatz einer Radiotherapie als „Bridging-Konzept“ beim r/r-DLBCL erweist sich sowohl als Monotherapie als auch in der Kombination mit einer Systemtherapie als effektive Strategie und im Vergleich mit systemtherapeutischen Konzepten als nicht unterlegen. Die RT ermöglicht eine rationale Überbrückung der Zeit zwischen Leukapherese und CAR-T-Zell-Infusion (ca. 4 Wochen).
- Lebensgefährdende Komplikationen nahmen nicht zu, wobei niedrig- und mittelgradige Toxizitäten nicht weiter aufgearbeitet wurden. Dies wäre angesichts der unterschiedlichen bestrahlten Körperregionen auch nicht sinnvoll gewesen. Trotzdem handelt es sich in der Studie um ein vulnerables Patientengut, denn immerhin erreichten 16,2 % der Patient*Innen die CAR-T-ZT nicht, meist infolge eines letalen Interimprogresses.
- Die Größe des hier dargestellten Patientenkollektivs übersteigt vergleichbare Analysen (12–31 Patient*Innen [6, 7]). Jedoch handelt es sich um eine nichtrandomisierte, monozentrische und lediglich retrospektive Auswertung mit den entsprechenden Einschränkungen. Die Gruppe der BT-Patient*Innen hatte schlechtere prognostische Charakteristika (Bulk, LDH, IPI, ECOG), sodass der initiale Prognoseunterschied in der Intention-to-treat-Analyse nicht überrascht. Dieser besteht jedoch nicht mehr beim Vergleich der Patient*Innen mit tatsächlich vorgenommener CAR-T-ZT. Entsprechend ist auch das verlängerte PFS in der alleinigen RT-Gruppe kritisch zu diskutieren, da Patient*Innen in dieser Kohorte gegenüber den anderen BT-Patient*Innen häufiger ein Ann-Arbor-Stadium I/II aufwiesen, im Median eine Therapielinie weniger durchliefen und damit putativ sensibler gegenüber einer erneuten Salvage-Therapie waren.
- Insgesamt bewirkt die Bridging-Strahlentherapie eine hohe lokale Kontrolle bzw. Ansprechrate [5], die sich auch in anderen Untersuchungen nachweisen ließ (80 % [7], 100 % [6]). Eine Rezidivanalyse von 31 r/r-DLBCL-Patient*Innen demonstrierte korrespondierend hierzu, dass bei 86 % ein „local failure“ nach CAR-T-ZT auftrat [8]. Besonders Stellen mit einem SUV_max_ ≥ 10 im PET-CT, einem Durchmesser von ≥ 5 cm oder ein extranodaler Befall disponieren für ein Lokalrezidiv, sodass diese für eine lokale RT präferenziell zu adressieren sind [8].
- Vor diesem Hintergrund ist die adäquate Felddefinition zu diskutieren. Die hier vorgestellte Arbeit plädiert für die Inklusion aller Krankheitsmanifestationen, wobei die Fallzahl (9 vs. 8 Patient*Innen) gering ist. Andere Arbeitsgruppen schlagen eine Involved-site-Radiotherapie [6] oder ein individuelles Vorgehen vor [7].
- Weiterhin bleiben die genaue Dosis und Fraktionierung in dieser Behandlungssituation eine individuelle Entscheidung. Wie in der Literatur [6, 7] besteht auch in dieser Analyse eine Tendenz zur (moderaten) Hypofraktionierung mit medianen Dosierungen von 20 bis 37,5 Gy. Diese ergeben sich aus der Notwendigkeit einer raschen Komplettierung der BT vor CAR-T-ZT. Ob hierdurch zusätzlich eine Immunmodulation oder Auslösung eines abskopalen Effekts induziert wird, bleibt offen. Entsprechende Dosiskonzepte werden auch durch die International Lymphoma Radiation Oncology Group (ILROG) empfohlen: 30 Gy à 3 Gy [9]. An unserem eigenen hämatoonkologischen Zentrum haben sich 36 Gy à 3 Gy ED etabliert, deren erhöhte biologische Wirksamkeit der Aggressivität der refraktären Erkrankung Rechnung trägt (α/β = 3 Gy EQD_2_ = 43,2 Gy; α/β = 10 Gy EQD_2_ = 39,0 Gy) in Analogie zu den Empfehlungen der ILROG für nicht transplantierbare r/r-DLBCL (45–55 Gy in Normofraktionierung; [10]).

## Fazit

Die dargestellte Arbeit weist auf eine mögliche neue Indikation der Radiotherapie in der Behandlung rezidivierter oder refraktärer DLBCL-Patienten hin. Der Einsatz der Bestrahlung als „Bridging-Konzept“ erscheint praktikabel und war nicht mit relevanter Toxizität verbunden.

Die präzisere Definition des Stellenwerts der Radiotherapie sowie deren geeignete Zielvolumina und Dosierungen müssen in künftigen Studien evaluiert werden.

*Michael Oertel und Hans Theodor Eich, Münster*
